# Association of Metabolic Syndrome with the Adiponectin to Homeostasis Model Assessment of Insulin Resistance Ratio

**DOI:** 10.1155/2015/607364

**Published:** 2015-10-18

**Authors:** Yu-Song Ding, Shu-Xia Guo, Ru-Lin Ma, Shu-Gang Li, Heng Guo, Jing-Yu Zhang, Mei Zhang, Jia-Ming Liu, Jia He, Yi-zhong Yan, Wen-Jie Zhang, Lie-Gang Liu

**Affiliations:** ^1^Department of Nutrition and Food Hygiene, Tongji Medical College, Huazhong University of Science and Technology, Wuhan 430030, China; ^2^Department of Public Health, Shihezi University School of Medicine, Shihezi, Xinjiang 832000, China; ^3^Department of Pathology and Key Laboratory of Xinjiang Endemic and Ethnic Diseases (Ministry of Education), Shihezi University School of Medicine, Shihezi, Xinjiang 832002, China

## Abstract

This study aimed at determining whether the adiponectin to HOMA-IR (A/H) ratio is associated with MetS and MetS components and comparing the diagnostic efficacy of adiponectin, HOMA-IR, and the A/H ratio in healthy, middle-aged participants. MetS was assessed in 1628 Kazakh participants (men, 768; women, 860). The associations between adiponectin, HOMA-IR, and the A/H ratio with the components of MetS and MetS were examined using logistic regression analysis and receiver operating characteristic (ROC) curves. Our results show that A/H ratio may be a better diagnostic marker for MetS than either HOMA-IR or adiponectin alone, and it may serve as an important biomarker to determine an increased risk for MetS in healthy middle-aged population.

## 1. Introduction

Metabolic syndrome (MetS) refers to several interrelated cardiometabolic risk factors including dysglycemia, obesity (particularly central adiposity), elevated blood pressure, elevated triglyceride (TG) levels, and low high-density lipoprotein cholesterol (HDL-C) levels [1, 2]. The prevalence of MetS is approximately 25% in adults, and it is increasing [3, 4]. MetS and its components are associated with an increased risk of type 2 diabetes and cardiovascular disease [5, 6]. The risks of heart disease, stroke, and diabetes are increased 1.5- to 3-fold in people with MetS compared with people without MetS [7]. As a result, MetS is now both public health and clinical problem [8]. Therefore, to decrease the incidence, there is a need to establish a suitable and sensitive screening marker to identify individuals at high risk for MetS.

The accumulated evidence indicates that insulin resistance (IR) with compensatory hyperinsulinemia is an important pathogenic factor for MetS [9, 10], although a precise mechanism linking a specific MetS component with IR is lacking [11, 12]. In epidemiological studies, the homeostasis model assessment-insulin resistance (HOMA-IR) acts as an important index of IR [13, 14].

Adiponectin, which is the most abundant circulating adipokine, is recognized as a critical regulator of insulin sensitivity [15, 16], tissue inflammation [17, 18], and lipid metabolism [19, 20]. Furthermore, a growing body of evidence suggests that decreased serum adiponectin is associated with most of the MetS components and therefore MetS [21, 22].

Hyperinsulinemia might have a negative impact on circulating adiponectin levels, thereby causing IR. HOMA-IR and adiponectin are thought to represent two different and opposite aspects of IR. The adiponectin concentration to HOMA-IR ratio (A/H ratio) is expected to be more sensitive than either parameter alone for the evaluation of MetS risk.

The A/H ratio as an index of MetS was first proposed in 2011 [23]. However, this study included an aged Japanese sample, and the analysis included markers that were measured only once. The association between the A/H ratio and MetS is yet to be confirmed, owing to limited evidence. Therefore, studies are needed to determine if there is a relationship between the A/H ratio and MetS and if this relationship is stronger than the individual parameters. This study aimed at determining whether the A/H ratio is associated with MetS and comparing the strength of the associations between MetS and adiponectin, HOMA-IR, and the A/H ratio.

## 2. Materials and Methods

### 2.1. Ethics Statement

The Institutional Ethics Review Board (IERB) at the First Affiliated Hospital of Shihezi University School of Medicine approved the study (IERB number SHZ2009LL05). Standard university hospital guidelines including informed consent, voluntary participation, confidentiality, and anonymity were followed. All participants provided written informed consent before participation.

### 2.2. Settings and Participants

The survey was conducted from 2009 to 2013 in Xinyuan County, Xinjiang, which is located approximately 4,400 km (2,739 miles) from Beijing; approximately 98% of the population is Kazakhs. Multistage (prefecture-county-township-village) stratified cluster random sampling was used to select the participants. At the beginning of the study, we chose the Yili prefecture based on the geographical distributions of the minority populations in Xinjiang. We randomly selected one county in Yili prefecture and one township from each county (Nalati Township in Xinyuan County). During the last stage, a stratified sampling method was used to select corresponding villages in each township (3 villages in Nalati Township). We interviewed local Kazakhs aged ≥ 18 years who had resided in the village for at least 6 months. We successfully interviewed a total of 1628 individuals (860 women and 768 men). Exclusion criteria included acute illness within the previous 2 weeks, currently taking medication, cancer, and pregnancy. The overall response rate was 87.0%.

### 2.3. Anthropometric Measurements and Laboratory Tests

Each participant was interviewed using a structured questionnaire to collect general and demographic information (age and sex) as well as cigarette smoking history (never smoked, ex-smoker, or current smoker). Waist circumference (cm) was measured midway between the lower rib and iliac crest. Weight (kg) and height (m) were measured with the participants in light clothing. Body mass index (BMI) was calculated as weight (kg) divided by the square of height (m^2^) and expressed as kg/m^2^. Casual blood pressure (BP) was measured 3 times after a 5 min rest in the sitting position using a mercury sphygmomanometer, and an average of 3 measurements was used for analyses. After the physical examination, a blood sample was drawn from the cubital vein in the morning after an overnight fast and was placed in tubes containing heparin sodium. The blood was centrifuged at 2000 rpm for 10 min, and plasma was then separated and stored at −70°C until analysis. Total cholesterol (TC), TG, low-density lipoprotein cholesterol (LDL-C), HDL-C, and fasting blood glucose (FBG) levels were measured using a biochemical autoanalyzer (Olympus AU 2700; Olympus Diagnostics, Hamburg, Germany) in the clinical laboratory at the First Affiliated Hospital of Shihezi University School of Medicine.

The circulating levels of interleukin- (IL-) 6 were determined using ELISA kits (Shanghai Westang Bio-Tech Co. Ltd.). Adiponectin levels were determined using ELISA kits (Phoenix Pharmaceuticals Inc., Belmont, CA, USA). All procedures described in the manufacturer's instructions were followed with quality control parameters within the expected range recommended by the manufacturer. Every tenth sample was duplicated on the same plate. The minimum detectable concentration of IL-6 kit is 0.8 pg/mL with the intra-assay CV < 3% and the interassay CV < 6.9%. The minimum detectable concentration of adiponectin kit is 0.15 ng/mL, with the intra-assay CV ranged from 3 to 6% and the interassay CV < 10%. Insulin level was measured by radioimmunoassay. The HOMA-IR index was defined as follows: fasting insulin (in micro-international units (*μ*IU) per mL) × FBG (in mM)/22.5 [7].

### 2.4. Definition of MetS

MetS was defined using the International Diabetes Federation (IDF) criteria [24], which include central obesity (waist circumference ≥ 90 cm in men or ≥ 80 cm in women, Chinese population waist circumference cutoffs [25]) plus any 2 of the following 4 factors: elevated TG level (>150 mg/dL or 1.69 mmol/L); reduced HDL-C (<40 mg/dL or 1.04 mmol/L in men; <50 mg/dL or 1.29 mmol/L in women); elevated systolic BP (≥130 mmHg) or diastolic BP (≥85 mmHg); and elevated FBG (≥100 mg/dL).

MetS was also defined using the revised National Cholesterol Education Program Adult Treatment Panel III (NCEP-ATP III) criteria [26], which have any three or more of the following: waist circumference ≥ 90 cm in men or ≥80 cm in women (Chinese population waist circumference cutoffs [25]); triglyceride level ≥ 150 mg/dL or taking medication for increased triglycerides; high-density lipoprotein cholesterol (HDL-C) level < 40 mg/dL or taking medication to improve HDL-C; systolic blood pressure ≥ 130 mmHg or diastolic blood pressure ≥ 85 mmHg or taking antihypertensive agent; fasting glucose level ≥ 100 mg/dL or taking blood glucose-lowering agent.

### 2.5. Statistical Analysis

Continuous variables are presented as mean ± standard deviation (SD) for clinical characteristics or median (interquartile range) for IL-6, adiponectin, and fasting insulin levels. These variables were compared using unpaired* t*-tests or Mann-Whitney* U* tests. The partial correlation coefficient was used to analyze the association between adiponectin, HOMA-IR, A/H ratio, and other continuous variables of interest, controlling for the effect of age. Multivariable logistic regression analysis with MetS as the dichotomous dependent variable was conducted to determine the association between adiponectin, HOMA-IR, A/H ratio, and MetS. The resulting odds ratios (ORs) and 95% confidence intervals (CIs) are reported. The receiver operating characteristic (ROC) analyses were used to describe the ability of the adiponectin, HOMA-IR, and A/H ratio to differentiate between subjects with and without metabolic syndrome. ROC analyses were also used to evaluate the difference in the contribution of the adiponectin, HOMA-IR, and A/H ratio to the risk levels of each component of MetS. All analyses were performed using SPSS v17.0 for Windows (SPSS Inc., Chicago, IL, USA). Differences with a* p* value of <0.05 were considered statistically significant.

## 3. Results

The characteristics of the study population, based on sex and presence of MetS, are provided in Table 1. IL-6 levels, insulin levels, HOMA-IR, and other anthropometric and metabolic characteristics were significantly greater in the MetS group than in the non-MetS group in both men and women (*p* < 0.05). In contrast, adiponectin levels, the A/H ratio, and HDL-C levels were significantly lower in the MetS group than in the non-MetS group (*p* < 0.05).

The correlations between the adiponectin levels, HOMA-IR, and A/H ratio and the risk factors of MetS are presented in Table 2. Adiponectin levels and the A/H ratio were negatively correlated with waist circumference, BMI, TC, TG, FBG, insulin, IL-6, and HOMA-IR (all *p* < 0.05). The correlation coefficients for BMI, waist circumference, TG, FBG, LDL, insulin, IL-6, and HOMA-IR with the A/H ratio were higher than those with adiponectin.

The multivariable adjusted ORs (95% CI) showed that the highest quartiles of adiponectin, HOMA-IR, and the A/H ratio were significantly associated with MetS, compared with the lowest quartiles (Table 3). In models I, II, and III, the adjusted ORs for MetS were higher with the A/H ratio than with adiponectin. In model III, which was adjusted for sex, age, smoking status, LDL-C, TC, and HDL-C, adiponectin (OR, 0.30; 95% CI, 0.19–0.46), HOMA-IR (OR, 3.82; 95% CI, 2.42–6.04), and A/H ratio (OR, 0.25; 95% CI, 0.15–0.40) remained significantly associated with MetS.

**Figure 1:**
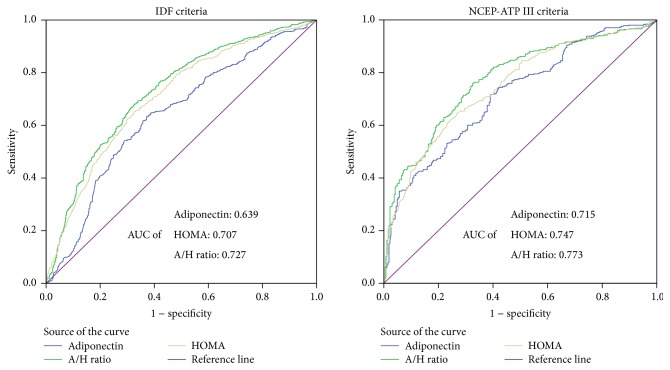
Comparison of predicting powers between adiponectin, HOMA-IR, and A/H ratio for difference metabolic syndrome criteria. HOMA-IR, homeostasis model assessment of insulin resistance; A/H ratio, adiponectin to homeostasis assessment-insulin resistance; ROC, receiver operating characteristic; AUC, area under the curve; IDF, International Diabetes Federation; NCEP-ATP III, National Cholesterol Education Program Adult Treatment Panel III.

Receiver operating characteristic (ROC) analysis was performed to detect the performance of the adiponectin, HOMA-IR, and A/H ratio as a diagnostic marker for MetS defined by the IDF and ATP III (Figure 1). As Figure 1 shows, the area under curve (AUC) of the A/H ratio, HOMA-IR, and adiponectin to detect MetS was 0.727, 0.707, and 0.639, respectively, by IDF criteria and 0.773, 0.747, and 0.715, respectively, by ATP III criteria. In addition we estimated that the best cutoff value for the A/H ratio to identify a risk of MetS was 2.10 (sensitivity, 0.68; specificity, 0.67), by IDF criteria. We estimated that the best cutoff value for the A/H ratio to identify a risk of MetS was 1.89 (sensitivity, 0.76; specificity, 0.67), by ATP III criteria.

The adjusted ORs (95% CI) and AUC to detect the MetS components are shown in Table 4. After adjustment for age, BMI, smoking status, and LDL-C, adiponectin, HOMA-IR, and the A/H ratio were all significantly associated with the MetS components. Except for BP, the ORs for the MetS components were lower for the A/H ratio than for adiponectin. Except for low HDL and abnormal glucose, the AUCs of the MetS components were higher for the A/H ratio than for adiponectin and HOMA-IR.

## 4. Discussion 

In this study, we found that the A/H ratio is more strongly associated with MetS and most of the MetS components than adiponectin. In addition, the A/H ratio showed greater predictive power than adiponectin and HOMA-IR for the risk of MetS. The A/H ratio is better at correctly classifying subjects with and without MetS than adiponectin or HOMA-IR alone.

Adiponectin is a multifunctional protein with pleiotropic insulin-sensitizing effects and is considered a key molecule in the pathogenesis of MetS [16, 27, 28]. In the present study, adiponectin levels were negatively correlated with waist circumference, BMI, TC, TG, FBG, insulin, IL-6, and HOMA-IR (all *p* < 0.05); when adjusted for sex, age, smoking status, and LDL-C, adiponectin remained significantly associated with MetS and MetS components. These findings are consistent with those of previous reports [21, 29]. Our previous results suggest that decreased adiponectin levels and HOMA-IR might be associated with IR and can predict the course of MetS [30].

The A/H ratio was significantly lower in the MetS group than in the non-MetS group (*p* < 0.05). Furthermore, the A/H ratio was significantly associated with MetS and MetS components after adjustment for sex, age, smoking status, and LDL-C. These results support the suggestion that the A/H ratio could be a powerful index for the evaluation of MetS [23]. However, there are few studies that have compared the strength of the associations between MetS and adiponectin and the A/H ratio or the ability of the A/H ratio to classify subjects with and without MetS. It is important to clarify the diagnostic power of adiponectin and the A/H ratio for future clinical use.

In our study, the A/H ratio showed a greater predicting power than adiponectin. For example, the correlation coefficients for BMI, waist circumference, TG, FBG, LDL, insulin, IL-6, and HOMA-IR with the A/H ratio were higher than those with adiponectin. Except for BP, the ORs for MetS and MetS components were lower for the A/H ratio than for adiponectin. We also conducted ROC analyses with the same participants using the IDF and updated ATP III definition for MetS, and the AUCs of the A/H ratio were higher than those for adiponectin and HOMA-IR by IDF and updated ATP III definition. In addition, except for low HDL and abnormal glucose, the AUCs of the MetS components were also higher for the A/H ratio than for adiponectin and HOMA-IR.

We performed the additional analysis to obtain the best cutoff for IDF and updated ATP III definition. We estimated that the best value for the A/H ratio to identify a risk of MetS was 2.10 (sensitivity, 0.68; specificity, 0.67), by IDF criteria. We estimated that the best cutoff value for the A/H ratio to identify a risk of MetS was 1.89 (sensitivity, 0.76; specificity, 0.67), by ATP III criteria. The A/H ratio has similar values of sensitivity and specificity with leptin/adiponectin ratio [25, 31].

These results could further explain our finding that the A/H ratio has a significant adjunctive contribution, beyond that of the adiponectin and HOMA-IR alone, to metabolic syndrome.

This study had several limitations. First, the cross-sectional design was not able to determine a causal relationship between MetS or its components and adiponectin HOMA-IR and A/H ratio. Second, we did not evaluate high molecular weight adiponectin, which is considered to be more useful than total adiponectin in evaluating the MetS and IR [31, 32]. Further investigation regarding the role of the ratio of high molecular weight adiponectin and HOMA-IR in MetS is needed.

In conclusion, to the best of our knowledge, this was the first large-scale population-based study to compare the diagnostic efficiency of adiponectin, HOMA-IR, and the A/H ratio in healthy middle-aged participants. We demonstrated that the A/H ratio can act as a marker of MetS and its components, serving as an important surrogate biomarker for MetS risk, and the A/H ratio contributed more to MetS than either HOMA-IR or adiponectin alone. As a result, this study provides useful information for clinicians to identify individuals at high risk of MetS. These results also show that the A/H ratio is helpful in understanding cardiometabolic diseases.

## Figures and Tables

**Table 1:** General characteristics of the study participants according to presence or absence of MetS.

Parameters	Men (*n* = 768)			Women (*n* = 860)		
	Without MetS (*n* = 504)	With MetS (*n* = 264)	*p*	Without MetS (*n* = 593)	With MetS (*n* = 267)	*p*
Anthropometric characteristics						
Age (y)	44.44 ± 13.98	50.34 ± 11.47	<0.01	40.17 ± 12.25	49.38 ± 11.48	<0.01
Waist circumference (cm)	83.07 ± 9.31	99.14 ± 8.17	<0.01	77.46 ± 9.67	91.52 ± 8.27	<0.01
BMI (kg/m^2^)	23.10 ± 3.09	28.60 ± 3.37	<0.01	22.60 ± 3.43	27.39 ± 3.65	<0.01
Systolic BP (mmHg)	126.80 ± 21.61	144.60 ± 20.34	<0.01	122.60 ± 19.89	144.73 ± 25.60	<0.01
Diastolic BP (mmHg)	81.59 ± 13.33	92.39 ± 12.12	<0.01	78.72 ± 12.26	92.03 ± 12.85	<0.01
Current smoker (*n* [%])	258 (51.29%)	214 (81.06%)	<0.01	231 (38.89%)	214 (80.15%)	<0.01
Metabolic characteristics						
Total cholesterol (mmol/L)	4.14 ± 0.98	4.75 ± 1.23	<0.01	4.13 ± 1.08	4.57 ± 1.28	<0.01
TG (mmol/L)	1.01 ± 0.48	2.06 ± 1.30	<0.01	0.93 ± 0.42	1.55 ± 0.87	<0.01
HDL cholesterol (mmol/L)	1.42 ± 0.42	1.38 ± 0.57	<0.01	1.60 ± 0.43	1.31 ± 0.49	<0.01
LDL cholesterol (mmol/L)	2.19 ± 0.69	2.53 ± 0.97	<0.01	2.05 ± 0.69	2.49 ± 0.82	<0.01
FBG (mmol/L)	4.47 ± 0.93	5.57 ± 1.47	<0.01	4.28 ± 0.79	5.19 ± 1.22	<0.01
Insulin (*μ*IU/dL)	10.80 (7.01–14.72)	14.95 (12.30–17.00)	<0.01	9.60 (7.20–15.50)	14.61 (8.40–17.50)	<0.01
HOMA-IR	2.05 (1.32–3.00)	3.31 (2.47–4.54)	<0.01	1.81 (1.37–3.08)	3.28 (1.77–4.31)	<0.01
Adiponectin (*µ*g/mL)	5.78 (4.18–6.63)	4.04 (2.67–5.65)	<0.01	6.98 (5.43–8.40)	5.70 (3.93–8.12)	<0.05
A/H ratio	2.43 (1.50–3.95)	1.08 (0.75–1.91)	<0.01	3.30 (2.08–5.00)	2.02 (1.00–3.57)	<0.01
IL-6 (pg/mL)	0.65 (0.16–1.39)	1.58 (1.18–1.97)	<0.01	0.53 (0.15–1.33)	1.60 (0.99–2.01)	<0.01

Values are expressed as means ± SD or number (%), if not stated otherwise. Median values of adiponectin, fasting insulin, IL-6, and HOMA-IR are presented (lower quartile-upper quartile).

SD, standard deviation; BMI, body mass index; TG, triglyceride; BP, blood pressure; HDL, high-density lipoprotein; LDL, low-density lipoprotein; FBG, fasting blood glucose; HOMA-IR, homeostasis model assessment of insulin resistance; IL-6, interleukin-6; A/H ratio, adiponectin to homeostasis assessment-insulin resistance.

**Table 2:** Partial correlation analysis among adiponectin, HOMA-IR, A/H ratio, and risk factors of MetS.

	A/H ratio	Adiponectin	HOMA-IR
Waist circumference	−0.220 (<0.001)	−0.212 (<0.001)	−0.210 (<0.001)
BMI	−0.259 (<0.001)	−0.254 (<0.001)	−0.222 (<0.001)
Systolic BP	−0.056 (0.025)	−0.121 (<0.001)	0.050 (0.043)
Diastolic BP	−0.085 (0.001)	−0.132 (<0.001)	0.071 (0.004)
Total cholesterol	−0.076 (0.002)	−0.089 (<0.001)	0.212 (<0.001)
TG	−0.172 (<0.001)	−0.016 (<0.001)	0.222 (<0.001)
HDL cholesterol	0.047 (<0.058)	0.047 (0.061)	−0.052 (0.037)
LDL cholesterol	−0.069 (<0.001)	−0.055 (0.026)	0.212 (<0.001)
FBG	−0.261 (<0.001)	−0.091 (<0.001)	0.398 (<0.001)
Insulin	−0.485 (<0.001)	−0.059 (0.017)	0.875 (<0.001)
IL-6	−0.159 (<0.001)	−0.082 (0.001)	0.151 (<0.001)
Adiponectin	0.618 (<0.001)	—	−0.112 (<0.001)
HOMA-IR	−0.521 (<0.001)	−0.112 (<0.001)	—
A/H ratio	—	0.618 (<0.001)	−0.521 (<0.001)

Values are age- and gender-adjusted Spearman correlation coefficients and *p* values for correlations of adiponectin, HOMA-IR, and A/H ratio with risk factors of MetS.

BMI, body mass index; BP, blood pressure; TG, triglyceride; HDL, high-density lipoprotein; LDL, low-density lipoprotein; FBG, fasting blood glucose; HOMA-IR, homeostasis assessment-insulin resistance; A/H ratio, adiponectin to homeostasis assessment-insulin resistance; MetS, metabolic syndrome.

**Table 3:** Odds ratios and 95% confidence intervals for the association between metabolic syndrome and various markers.

	Odds ratio (95% CI)		
	Model I	Model II	Model III
Adiponectin			
1st quartile	1	1	1
2nd quartile	0.59 (0.44–0.79)	0.65 (0.44–0.95)	0.66 (0.44–0.99)
3rd quartile	0.29 (0.21–0.40)	0.58 (0.37–0.89)	0.60 (0.38–0.95)
4th quartile	0.28 (0.20–0.40)	0.34 (0.23–0.51)	0.30 (0.19–0.46)
HOMA-IR			
1st quartile	1	1	1
2nd quartile	1.63 (1.11–2.32)	1.62 (1.04–2.52)	1.72 (1.08–2.73)
3rd quartile	2.88 (2.02–4.10)	2.05 (1.33–3.16)	1.83 (1.16–2.88)
4th quartile	7.36 (5.19–10.46)	3.88 (2.52–5.97)	3.82 (2.42–6.04)
A/H ratio			
1st quartile	1	1	1
2nd quartile	0.30 (0.22–0.40)	0.53 (0.35–0.80)	0.52 (0.33–0.83)
3rd quartile	0.25 (0.18–0.16)	0.41 (0.28–0.60)	0.43 (0.28–0.66)
4th quartile	0.11 (0.08–0.16)	0.23 (0.15–0.35)	0.25 (0.15–0.40)

CI, confidence interval; HOMA-IR, homeostasis model assessment of insulin resistance; A/H ratio, adiponectin to homeostasis assessment-insulin resistance; MetS, metabolic syndrome; IL-6, interleukin-6.

Model I: adjusted for sex and age.

Model II: adjusted for sex, age, body mass index, smoking status, and low-density lipoprotein cholesterol.

Model III: adjusted for sex, age, body mass index, smoking status, low-density lipoprotein cholesterol, total cholesterol, and high-density lipoprotein cholesterol.

**Table 4:** Odds ratios (95% CI) and ROC analysis for the association between each component of MetS and markers.

	Adiponectin (Q4 versus Q1)		HOMA-IR (Q4 versus Q1)		A/H ratio (Q4 versus Q1)	
	OR^*∗*^ (95% CI)	AUC (SE)	OR^*∗*^ (95% CI)	AUC (SE)	OR^*∗*^ (95% CI)	AUC (SE)
Abdominal obesity	0.37 (0.27–0.51)	0.58 (0.01)	2.74 (2.01–3.72)	0.61 (0.01)	0.20 (0.13–0.31)	0.63 (0.01)
High triglycerides	0.42 (0.28–0.65)	0.64 (0.02)	4.35 (2.82–6.71)	0.67 (0.02)	0.20 (0.13–0.31)	0.69 (0.02)
High blood pressure	0.33 (0.24–0.46)	0.61 (0.01)	1.55 (1.14–2.10)	0.55 (0.01)	0.44 (0.33–0.59)	0.61 (0.01)
Low HDL	0.71 (0.51–0.99)	0.51 (0.02)	1.74 (1.25–2.42)	0.57 (0.02)	0.50 (0.36–0.69)	0.55 (0.02)
Abnormal glucose	0.42 (0.28–0.63)	0.63 (0.02)	10.08 (6.32–16.06)	0.75 (0.02)	0.14 (0.09–0.23)	0.73 (0.02)

CI, confidence interval; AUC, area under the curve; SE, standard error; A/H ratio, adiponectin to homeostasis assessment-insulin resistance; MetS, metabolic syndrome; Q4, highest quartile; Q1, lowest quartile.

^*∗*^Adjusted for sex, age, smoking status, and LDL cholesterol.

## Abbreviations

BMI: Body mass index
BP: Blood pressure
TC: Total cholesterol
TG: Triglyceride
HDL: High-density lipoprotein
LDL: Low-density lipoprotein
FBG: Fasting blood glucose
HOMA-IR: Homeostasis assessment-insulin resistance
A/H ratio: Adiponectin to homeostasis assessment-insulin resistance
MetS: Metabolic syndrome
ORs: Odds ratios
CIs: Confidence intervals
ROC: Receiver operating characteristic
AUC: Area under the curve
IL-6: Interleukin-6.
